# Regenerative Endodontic Procedure of a Mature Anterior Tooth With Open Apex and Chronic Apical Abscess: A Case Report With 4‐Years Follow‐Up

**DOI:** 10.1002/ccr3.70911

**Published:** 2025-09-22

**Authors:** Murilo Priori Alcalde, Raimundo Sales de Oliveira Neto, Estela Franzolin Pires de Almeida, Renan Diego Furlan, Paulo Roberto Jara de Souza, Rodrigo Ricci Vivan, Pablo Andres Amoroso‐Silva, Marco Antônio Húngaro Duarte, Guilherme Ferreira da Silva

**Affiliations:** ^1^ Department of Dentistry, Endodontics and Dental Materials, Bauru Dental School University of São Paulo Bauru Brazil; ^2^ Department of Dentistry Unisagrado Bauru Brazil; ^3^ Department of Restorative Dentistry Universidade Estadual de Londrina Londrina Paraná Brazil

**Keywords:** endodontics, pulp necrosis, regenerative, revascularization

## Abstract

This case report describes the successful treatment of a mature anterior tooth with an open apex and chronic apical abscess using a regenerative endodontic procedure. A 36‐year‐old man with a history of trauma to tooth 11 at age 10 presented with incomplete root development and an asymptomatic chronic apical abscess. Cone beam tomography revealed a periradicular lesion with buccal bone plate loss. After biomechanical preparation using K‐type files and irrigation with 1% sodium hypochlorite, calcium hydroxide was placed as intracanal medication for 14 days. Bleeding was induced by overinstrumenting the periapical tissues with a #25 K‐file, and a bioceramic material was placed over the blood clot, followed by a composite resin coronal seal. At the 4‐year follow‐up, the patient was asymptomatic, with complete bone plate formation and no periradicular lesion or root resorption, although no hard tissue formation or changes in root canal morphology were observed.

**Summary**

- Regenerative endodontics can successfully treat mature teeth with open apices and apical periodontitis, promoting periapical healing and partial pulp sensitivity recovery.
- While root lengthening/thickening may not occur, infection resolution and bone regeneration are achievable.
- Long‐term follow‐up is essential to assess stability, though age may limit regenerative potential.

## 1 Introduction

The treatment of pulp necrosis in teeth with open apices poses significant challenges due to factors like wide root canals, thin dentinal walls, and shortened roots [1]. Some authors have proposed regenerative endodontic procedures (REP) as an alternative for the treatment of mature teeth with open apex [2, 3, 4]. The aim of REP is to promote root development, increase root length, thicken the dentine walls, and achieve apical closure [5]. It is believed that REP may reduce the risk of tooth fracture, increasing its longevity [2, 3].

The success rate of REP ranges from 83% to 93% [6]. Several factors are essential for the success of REP, including control of endodontic infection, blood clot formation, stem cells, and activators [7]. However, the composition and concentration of the clot are unpredictable, and this uncertainty increases with age [2, 3, 4, 5].

Several case reports describe the use of REP in immature teeth with necrotic pulp in adult patients [3, 4, 5]. REP in mature teeth may present additional challenges due to the absence of stem cells from the apical papilla [8]. Therefore, certain conditions should be considered before treatment. The apical diameter should be at least 0.5 mm to allow for sufficient revascularization potential and to avoid degenerative changes in vessels and nerves over time [7]. Low levels of angiogenesis can affect the pulp–dentin complex, triggering calcifications and reducing dentin apposition [9]. Also, the ability of multipotent stem cells to replace damaged tissues tends to decrease [9]. Thus, the endodontist must perform a thorough clinical evaluation to select the most appropriate and predictable endodontic procedure for immature teeth in adult patients.

While REPs are well‐documented in immature teeth, their application in mature teeth—particularly in adult patients—remains less explored [2, 3, 4, 5, 8]. This case report describes the successful management of a 36‐year‐old patient with a mature central incisor (tooth 11) presenting both an open apex and chronic apical abscess, followed for 4 years. The extended follow‐up period provides valuable insights into the long‐term behavior of REP‐treated mature teeth in adults, addressing current gaps in understanding the procedure's viability in such challenging cases [3]. Our findings underscore the importance of careful case selection when applying regenerative approaches to mature teeth with open apices, while contributing to the growing evidence supporting REPs in nontraditional clinical scenarios.

## 2 Case History/Examination

**FIGURE 1:**
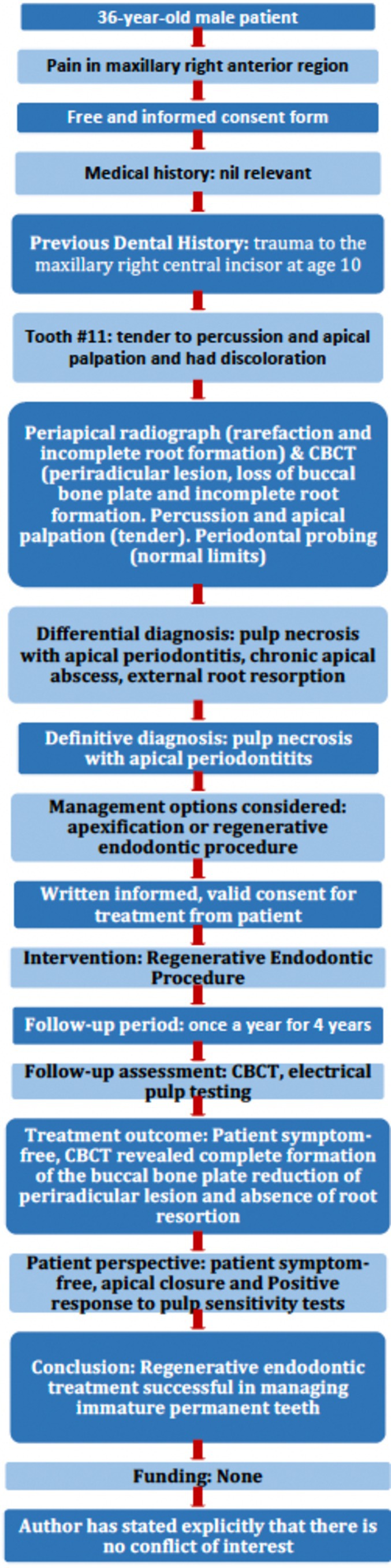
PRICE 2020 flowchart.

This case report has been written according to Preferred Reporting Items for Case reports in Endodontics (PRICE) 2020 guidelines—Figure 1 [10].

**FIGURE 2:**
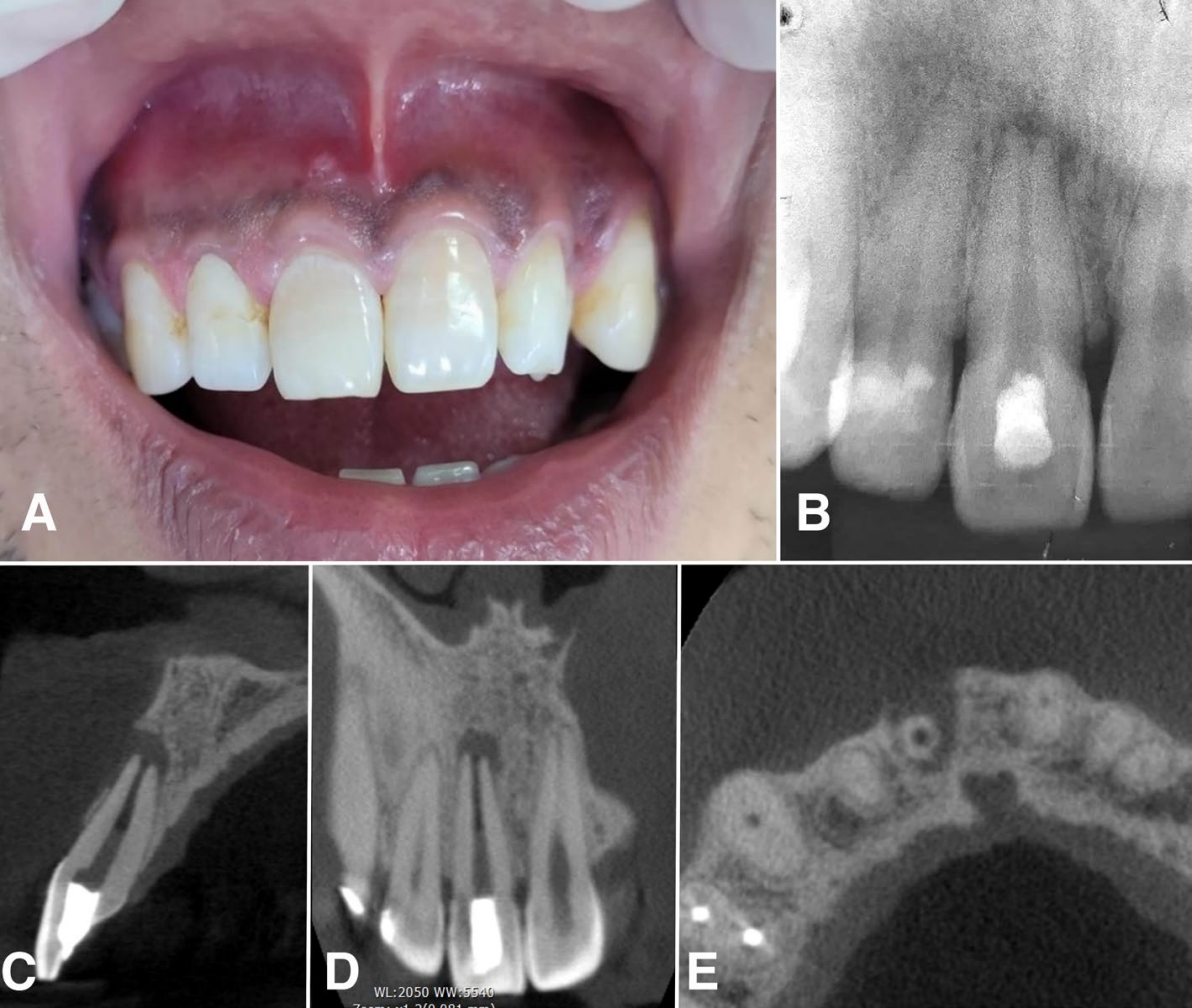
(A) Clinical aspect of maxillary right central incisor (tooth #11, FDI notation). A sinus tract is also visible a sinus tract on the apical gingiva. (B) Periapical radiograph of tooth #11 showing temporary restoration, apical lesion, and open apex. Preoperative CBCT sagittal (C), coronal (D), and axial views (E), showing loss of the buccal cortical plate (C and D), incomplete root formation, and apical lesion (C, D, and E).

A 36‐year‐old man attended the dental office with a chief complaint of pain in the maxillary right anterior region. Intraoral examination revealed a sinus tract present over the maxillary right central incisor—tooth #11, FDI notation—(Figure 2A)—which was tender to percussion and apical palpation. No tooth discoloration was observed clinically. The patient reported that five days prior to presenting to the dental office, coronal access was performed at the public health system's emergency service due to painful symptoms.

## 3 Differential Diagnosis, Investigation and Treatment

Periapical radiograph revealed a periradicular rarefaction around the apex of tooth #11 and incomplete root formation (Figure 2B). The patient reported a history of trauma to this tooth at the age of 10. A CBCT scan was taken and showed a periradicular lesion with concomitant loss of the buccal bone plate and incomplete root formation (Figure 2C–E), classified as 4D according to the Estrela et al. [11] periapical index, with a diameter of 4.6 mm and destruction of the periapical cortical bone. So, the diagnosis for tooth #11 was chronic apical abscess, and REP was considered as treatment. Other possible differential diagnoses include periapical cyst, periapical granuloma, external root resorption, or a developmental root anomaly due to the history of trauma, sinus tract, and radiographic findings.

After the patient's consent was obtained, the treatment was carried out in two sessions. Firstly, the patient was anesthetized with 2% lidocaine containing 1:100,000 epinephrine, and rubber dam isolation was placed. Coronal access was made, and then the root canal was irrigated with 10 mL of 1% sodium hypochlorite (NaOCl). Working length (WL) was established at 0.0 mm from the apex using an apex locator and a K‐File #50. A #30 K‐File was used in gentle circumferential motion to displace the necrotic tissue, and irrigation was conducted with 50 mL of 1% NaOCl. EDTA 17% was left in the canal for approximately 3 min, followed by a final flush with saline solution. The canal was dried with #50 sterile paper points. A calcium hydroxide paste was used as intracanal medication [12, 13], and the cavity was temporarily sealed with glass ionomer cement (SSWhite, Rio de Janeiro, Brazil).

**FIGURE 3:**
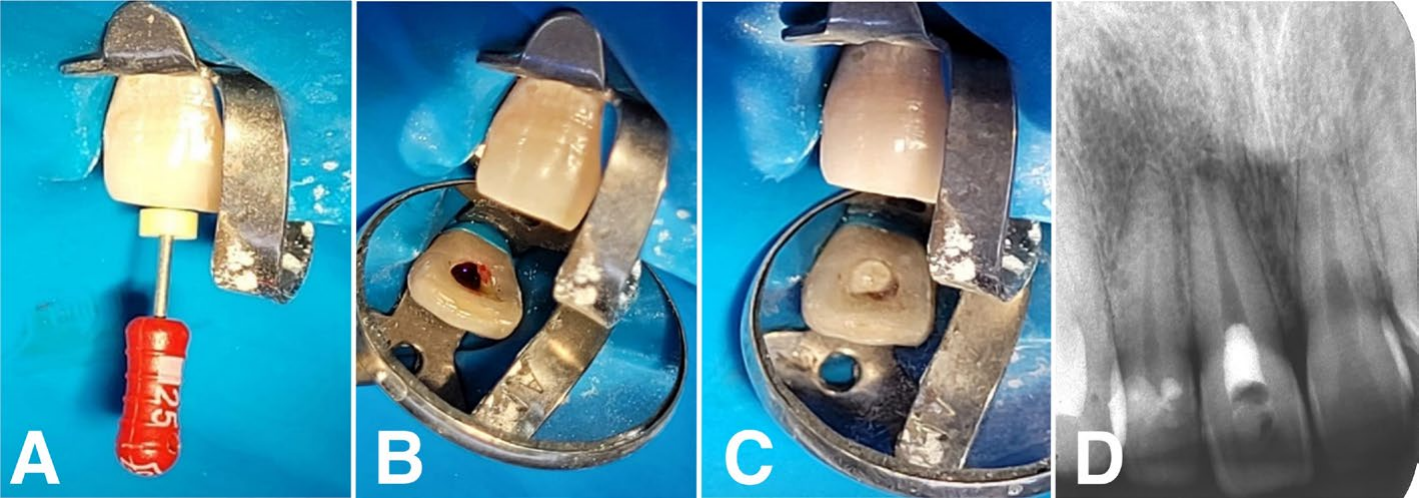
(A) A #25 K‐File introduced in the canal 2 mm beyond the root canal length to promote bleeding; (B) Intracanal bleeding and blood clot formation at the level of the cementoenamel junction (CEJ); (C) Clinical and (D) radiographic images of bioceramic material placed over the blood clot, forming a 3 mm plug.

After 14 days, the tooth remained asymptomatic, and the sinus tract was healed. The patient was anesthetized with 3% mepivacaine without epinephrine, and rubber dam isolation was performed. After removing the temporary seal, 10 mL of 1% NaOCl was used to remove the calcium hydroxide paste, followed by 5 mL of sterile saline solution. All irrigation procedures were performed using a 30‐G needle 3 mm short of the WL. Next, the canal was dried with sterile paper points, and a #25 K‐File was gently introduced 2 mm beyond the apical foramen (Figure 3A) in accordance with AAE regenerative endodontic guidelines [14] to intentionally stimulate bleeding into the canal space (Figure 3B,C) up to the cementoenamel junction (CEJ) level. Then, Sealer Plus BC powder‐liquid (MKLife, Porto Alegre, Brazil) was prepared and placed over the blood clot, forming a 4 mm plug (Figure 3D). The access cavity was sealed with glass ionomer cement and composite resin (3M, Sumaré, Brazil).

## 4 Outcome and Follow‐Up

**FIGURE 4:**
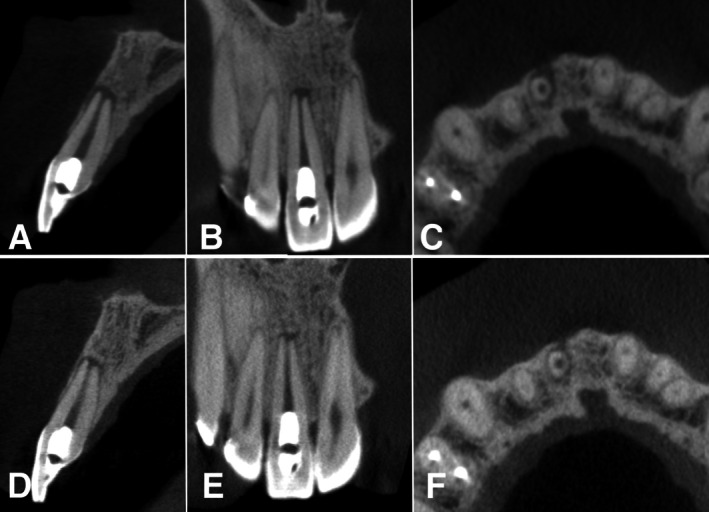
CBTC sagittal (A), coronal (B), and axial (C) images after one year of the endodontic regeneration procedure. Partial formation of the buccal bone plate in the sagittal cross‐section (A), reduction in the periradicular lesion in the coronal and axial cross‐sections (B and C). After 4 years, the new CBCT images show complete formation of the buccal bone plate (D); both the coronal (E) and axial (F) cross‐sections show almost complete healing of the apical lesion.

The patient returned for radiographic follow‐up every 3 months. A CBCT was performed at 12 months and images showed partial formation of the buccal bone plate in the sagittal cross‐section (Figure 4A), significant reduction in the periradicular lesion (Figure 4B,C). Also, the patient reported a mild sensitivity to electrical pulp testing. The 4‐year CBCT follow‐up exam revealed complete formation of the buccal bone plate (Figure 4D), resolution of the periapical radiolucency, and absence of root resorption (Figure 4E,F). Additionally, the patient still reported mild sensitivity to electrical pulp testing. No increase in root canal wall thickness is visible (B vs. E).

**FIGURE 5:**
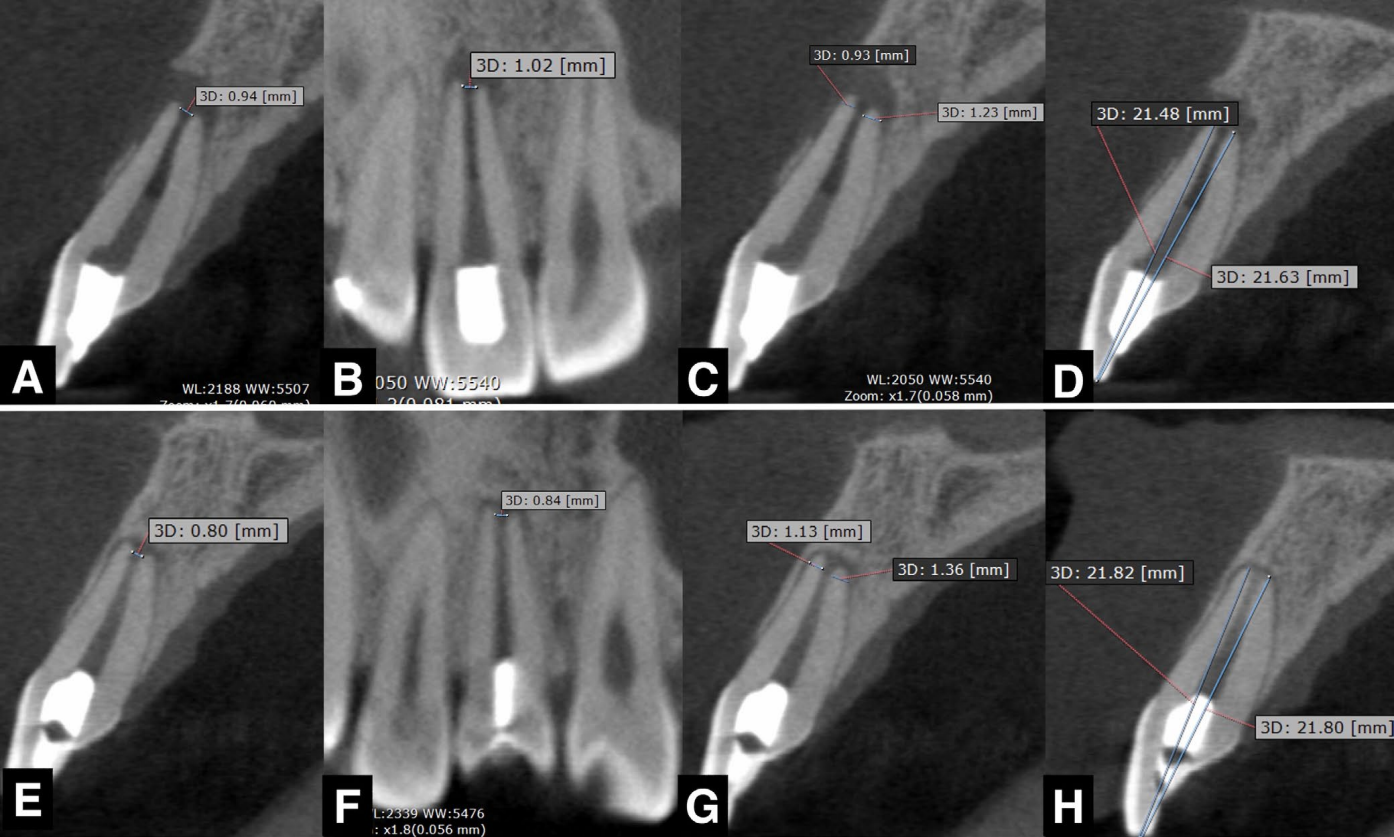
CBCT measurements of tooth #11. Pre‐treatment: (A) Foraminal diameter (sagittal view) = 0.94 mm; (B) Foraminal diameter (coronal view) = 1.02 mm; (C) Dentin thickness (buccal = 0.93 mm, palatal = 1.23 mm); (D) Root length = 21.48–21.63 mm. Post‐treatment: (E) Foraminal diameter (sagittal view) = 0.80 mm; (F) Foraminal diameter (coronal view) = 0.84 mm; (G) Dentin thickness (buccal = 1.13 mm, palatal = 1.36 mm); (H) Root length = 21.82–21.80 mm.

For a better assessment and comparison of the outcomes of the regenerative endodontic procedure, CBCT images were analyzed. Parameters assessed included apical canal diameter (mesiodistal and buccopalatal dimensions), dentin thickness (mesiodistal and buccopalatal dimensions), buccal bone loss extent, and periapical lesion size [11] pre‐ and post‐treatment. Measurements were obtained using the proprietary measurement tool in OnDemand Dental Software (Cybermed Inc., Seoul, South Korea) (Table 1). While the most prominent tomographic findings showed the reduction of the periapical lesion (from stage 4D pre‐treatment to stage 0 post‐treatment) [11] and buccal bone loss, some minor changes were observed in apical diameter and dentin thickness (Figure 5). These outcomes, particularly the complete healing observed, demonstrate the efficacy of REP in adult patients.

**TABLE 1:** The values of apical diameter, root length, dentin thickness, extent of buccal bone loss, and extent of periapical lesion of the tooth assessed using CBCT images before and after endodontic regeneration procedure.

	Apical canal diameter		Root length		Dentin thickness B‐P		Dentin thickness M‐D		Bone loss	Lesion extension	
	B‐P	M‐D	B	P	B	P	M	D	B	B‐P	M‐D
Initial	0.94	1.02	21.48	21.63	0.93	1.23	0.96	1.10	5.71	4.60	4.13
Final	0.80	0.84	21.82	21.80	1.13	1.36	1.03	1.10	Healed	Healed	Healed

Abbreviations: B, buccal; B‐P, bucco‐palatal direction; D, distal; M, mesial; M‐D, mesio‐distal direction; P, palatal.

## 5 Discussion

REP has been widely used to treat immature teeth in young patients [2, 3], and it also represents a viable option for treating mature teeth in adults with apical periodontitis [4, 8]. After thorough discussion of alternative treatment options (including apexification with calcium hydroxide or MTA, and intentional replantation), REP was selected through shared decision‐making between the clinician and patient, considering the tooth's favorable anatomical conditions for regeneration and the procedure's biological advantages [12]. Previous studies stated that the minimum apical diameter should be 0.5 mm to allow for adequate blood supply and angiogenesis [5, 8]. The authors used REP because the apical diameter was compatible with a #50 K file, as determined using an apex locator.

It is recommended to use 1.5% NaOCl for REP, as this lower concentration maintains its bacterial effectiveness while reducing cytotoxicity and minimizing harm to stem cells [12, 13]. Thus, the authors chose to use 1% NaOCl during the procedure. Additionally, 17% EDTA was used after instrumentation in both the first and second appointments to promote the release of endogenous growth factors from the dentin, enhance stem cell attachment, and support stem cell differentiation [14].

Triple antibiotic paste (TAP), double antibiotic paste (DAP), and calcium hydroxide paste (Ca(OH)_2_) [9] are strongly recommended for REPs [9, 14]. However, Ca(OH)_2_ does not cause tooth discoloration, has lower cytotoxicity, promotes greater survival and proliferation of stem cells, and stimulates the release of growth factors [12, 15]. A meta‐analysis found that Ca(OH)_2_ induced a higher percentage of apical closure [16]. However, TAP presents greater antibacterial capacity [17].

During the second appointment, intra‐canal bleeding was induced to create a blood clot as a scaffold, promoting the release of growth factors and stem cells from the apical region into the root canal [3, 5]. Intra‐canal bleeding can be influenced by severe destruction of periapical tissues, a persistent inflammatory response, and the use of local anesthetics containing epinephrine [3]. A thorough clinical evaluation was conducted during the second appointment to verify the absence of pain or signs of persistent infection. Also, no anesthetics containing epinephrine were used.

Bioceramic material with zirconium oxide was chosen for this case due to its practical advantages over MTA, while maintaining similar clinical outcomes [18]. This type of material offers faster setting time, easier handling, and no risk of tooth discoloration, crucial for anterior teeth. Also, its proven ability to stimulate mineralized tissue formation makes it an excellent choice for REP [19].

Although the patient's age may negatively affect blood supply and intracanal bleeding [20, 21], suitable intra‐canal bleeding was successfully achieved. The success criteria for REPSs include the absence of inflammation, healing of pre‐existing bony lesions in the periapical tissues, increased root canal length and wall thickness, absence of inflammatory resorption, and a positive response to pulp sensibility testing [4, 6, 7]. Reported outcomes for regenerative procedures vary across studies, with success rates ranging from 76.47% [22] to 93% [23]. Root length changes show variability, from minimal increases (8.55% ± 8.97% in apexification cases [22]) to more significant growth (71.43% in successful revascularization cases [23]). Similarly, dentinal wall thickening ranges from 13.75% ± 19.91% [22] to 57%–72.6% [23], depending on case selection and evaluation criteria. In adult patients, narrower apical pathways can limit blood supply, stem cell migration, and angiogenesis compared to immature teeth [20, 21], which may explain the absence of increased root canal length and dentin wall thickness in this case. The 12‐month follow‐up demonstrated a significant reduction of the periapical radiolucency, with no observed changes in root canal length or wall thickness. At a 4‐year follow‐up, complete bone healing was observed, and the patient showed sensitivity to electrical pulp testing, demonstrating the success of the therapy.

While REPs show promising outcomes, potential complications and long‐term uncertainties warrant consideration. The risk of reinfection persists due to possible residual bacteria or scaffold instability [24]. Calcific metamorphosis and internal resorption are concerns, particularly as REP‐treated teeth lack an odontoblast layer, making them vulnerable to clastic cell activity via osteopontin‐mediated pathways [24, 25].

Current advances in pulp regeneration incorporate stem cell therapy and scaffold‐based techniques [4]. These three‐dimensional biomaterials aim to replicate the natural extracellular matrix, requiring optimal biocompatibility and mechanical stability. As platelet‐rich plasma has shown promise as a natural scaffold in clinical applications, the necessity of scaffolds for tissue repair remains debated [26, 27]. Further research with standardized protocols, larger cohorts, and long‐term monitoring is needed to validate these innovative treatments.

## 6 Conclusion

Although there was no increase in root canal length and/or dentin wall thickness, REP achieved complete bone healing and restored mild sensitivity to electrical pulp testing. Future randomized clinical trials should investigate how patient age influences REP success rates, while long‐term studies are needed to assess the stability of pulp testing responses and tooth survival over extended periods.

## Author Contributions

**Murilo Priori Alcalde:** investigation, writing – original draft. **Raimundo Sales de Oliveira Neto:** data curation, writing – original draft, writing – review and editing. **Estela Franzolin Pires de Almeida:** writing – review and editing. **Renan Diego Furlan:** writing – review and editing. **Paulo Roberto Jara de Souza:** writing – review and editing. **Rodrigo Ricci Vivan:** writing – review and editing. **Pablo Andres Amoroso‐Silva:** writing – review and editing. **Marco Antônio Húngaro Duarte:** writing – review and editing. **Guilherme Ferreira da Silva:** supervision.

## Disclosure

The authors have nothing to report.

## Ethics Statement

The authors have nothing to report.

## Consent

Written informed consent was obtained from the patients to publish this report in accordance with the journal's patient consent policy.

## Conflicts of Interest

The authors declare no conflicts of interest.

## Data Availability

The data that support the findings of this study are available from the corresponding author upon reasonable request.
